# CX3CR1 and malignant progression of glioma

**DOI:** 10.18632/aging.203536

**Published:** 2021-09-12

**Authors:** Sungho Lee, Benjamin Deneen, Ganesh Rao

**Affiliations:** 1 Department of Neurosurgery, Baylor College of Medicine, Houston, TX 77030, USA

**Keywords:** glioma, glioblastoma multiforme, chemokine, fractalkine, malignant progression

The varying period of time before an indolent low-grade glioma (LGG) inexorably transforms into a high-grade glioma (HGG) provides a unique opportunity for intervention. While recent clinical trials have demonstrated a clear survival benefit with early treatment of LGGs rather than watchful waiting, there is still a need to identify prognostic molecular markers that predict malignant transformation [1,2]. Although LGGs are often diagnosed in younger patients, as patients age, genetic instability and other changes in the tumor microenvironment can promote malignant progression to HGG. The driver mutations that define LGGs are well-established; however, genetic alterations that mediate recurrence and malignant transformation are essentially stochastic [3]. Therefore, identifying key shapers of the tumor microenvironment, rather than tumor-specific events, may provide a more consistent prognostic information and better therapeutic targets. In particular, tumor associated microglia and macrophages (TAMs) are immunosuppressive and proangiogenic, impeding effector anti-tumor lymphocyte responses and supporting invasion and growth of gliomas. Their pivotal role in malignant transformation of LGGs are only recently beginning to be understood.

CX3CR1 is a chemokine receptor expressed by TAMs and tumor cells. Its cognate ligand, CX3CL1 or fractalkine, can exist in a membrane-anchored or soluble form, and mediates adhesion or chemotaxis, respectively. In the CNS, CX3CL1-CX3CR1 signaling has been implicated in regulation of microglial recruitment and function in various disease contexts including stroke, Parkinson’s disease, Alzheimer’s disease [4]. CX3CR1 deficiency also delayed the formation of low-grade optic gliomas in a mouse model of neurofibromatosis type 1, in association with reduced microglia numbers [5]. Interestingly, *CX3CR1* has a common polymorphism in which valine at amino acid position 249 is substituted by isoleucine (V249I), conferring a reduced binding affinity to CX3CL1. In a previous study, CX3CR1 V249I polymorphism conferred a significant survival benefit in glioblastoma, in association with reduced numbers of TAMs, although the mechanistic details were incompletely explored [6].

Given the more protracted natural history, we hypothesized that the impact of the *CX3CR1* V249I polymorphism on survival may be more sizeable in LGGs compared to glioblastomas. Indeed, in our cohort of 90 patients with LGGs that ultimately progressed to HGGs, *CX3CR1* V249I polymorphism conferred a strong survival benefit, extending median overall survival by 5 years and progression-free survival by nearly 2 years [7]. This effect was especially evident in 1p19q deleted tumors, where the overall survival was doubled. Similar to Rodero et al., the numbers of M2 polarized, CD204 positive TAMs were selectively reduced in patients with the *CX3CR1* V249I polymorphism. Correspondingly, microvessel density was also lower, reflecting reduced angiogenesis.

Bulk tumor RNA sequencing data were examined for *CX3CR1* genotype dependent alterations. *CX3CR1* V249I polymorphism was associated with lower transcript levels of CCL2, implicated in TAM recruitment, and MMP9, involved in invasion and angiogenesis. Interestingly, these effects were at least in part mediated through the impact of the *CX3CR1* V249I polymorphism on the tumor cells. Several patient-derived glioblastoma stem cell (GSC) lines expressed CX3CR1 at the surface. GSCs derived from patients without the polymorphism responded to CX3CL1 stimulation by upregulating CCL2, whereas this response was not seen in GSCs from patients with the *CX3CR1* V249I polymorphism.

Next, these effects of intratumoral CX3CL1-CX3CR1 signaling were recapitulated in a PDGFB-driven mouse model of glioma. Co-expression of human CX3CL1 and CX3CR1 (wild-type) within tumors led to worsened survival and higher histological grade. Further characterization demonstrated increased density of TAMs and microvessel density as well. Taken together, these data suggest that intratumoral CX3CL1-CX3CR1 signaling promotes a proangiogenic and immunosuppressive microenvironment through generation of CCL2 and MMP9, which is abrogated in the presence of CX3CR1 V249I polymorphism.

In the CNS, CX3CR1 signaling has been mostly investigated in the context of regulating microglial phenotypes. However, it is also expressed in a wide variety of tumors outside the CNS and promote proliferation, invasion and survival. Our study is the first to characterize the important role for CX3CR1 signaling within the glioma cells to promote an immunosuppressive and pro-angiogenic microenvironment. These results of course do not rule out the likely contribution of microglial CX3CR1 signaling; but intratumoral CX3CR1 signaling was convincingly demonstrated through CX3CL1 mediated CCL2 upregulation in GSCs as well as characterization of phenotypes in CX3CL1/CX3CR1 co-expressing mouse models. Broadly, our data highlight the complex, intertwined nature of the interactions between the tumor and the microenvironment and how gliomas hijack normal signaling pathways. Full understanding of CX3CR1 signaling will require the resolution of single-cell sequencing in human tumors and use of cell-type-specific genetic manipulations in animal models – both of which are currently ongoing in our laboratory.

A recent clinical trial demonstrated the clear survival advantage of adjuvant chemotherapy and radiation compared to radiation alone following surgical resection of LGGs [2]. However, despite the subsequent paradigm shift toward more aggressive treatment of LGGs, it is clear that a blanket approach of surgical resection followed by adjuvant chemoradiation may not be appropriate for all tumors. Therefore, prognostic molecular markers, such as the *CX3CR1* V249I polymorphism, may play an important role in individualizing therapy, reserving maximal treatment for those LGGs which are likely to behave more aggressively.
